# Plasma From Older Children in Malawi Inhibits *Plasmodium falciparum* Binding in 3-Dimensional Brain Microvessels

**DOI:** 10.1093/infdis/jiae315

**Published:** 2024-06-14

**Authors:** Fatou Joof, Ruoqian Hu, Alex Saidi, Karl B Seydel, Lauren M Cohee, Ying Zheng, Joseph D Smith

**Affiliations:** Center for Global Infectious Disease Research, Seattle Children's Research Institute, Seattle, Washington, USA; Department of Bioengineering, University of Washington, Seattle, Washington, USA; Blantyre Malaria Project, Kamuzu University of Health Sciences, Blantyre, Malawi; Blantyre Malaria Project, Kamuzu University of Health Sciences, Blantyre, Malawi; Department of Osteopathic Medical Specialties, College of Osteopathic Medicine, Michigan State University, East Lansing, Michigan, USA; Center for Vaccine Development and Global Health, University of Maryland School of Medicine, Baltimore, Maryland, USA; Department of Bioengineering, University of Washington, Seattle, Washington, USA; Center for Global Infectious Disease Research, Seattle Children's Research Institute, Seattle, Washington, USA; Department of Pediatrics, School of Medicine, University of Washington, Seattle, Washington, USA

**Keywords:** cerebral malaria, Malawi, adhesion inhibition, antibody response, 3D brain microvessel

## Abstract

A hallmark of cerebral malaria is sequestration of *Plasmodium falciparum*-infected erythrocytes (IEs) in the brain microcirculation. Antibodies contribute to malaria immunity, but it remains unclear whether functional antibodies targeting parasite-expressed ligand can block cytoadhesion in the brain. Here, we screened the plasma of older children and young adults in Malawi to characterize the antibody response against the *P. falciparum*-IE surface and used a bioengineered 3-dimensional (3D) human brain microvessel model incorporating variable flow dynamics to measure adhesion-blocking responses. We found a strong correlation between surface antibody reactivity by flow cytometry and reduced *P. falciparum*-IE binding in 3D microvessels. Moreover, there was a threshold of surface antibody reactivity necessary to achieve robust inhibitory activity. Our findings provide evidence of the acquisition of adhesion-blocking antibodies against cerebral binding variants in people exposed to stable *P. falciparum* transmission and suggest the quality of the inhibitory response can be influenced by flow dynamics.

## Teaser

In highly endemic *Plasmodium falciparum* regions, older children acquire immunity that protects against severe malaria. This study used 3-dimensional human brain microvessels to demonstrate that older Malawian children acquire adhesion-blocking antibodies that may contribute to cerebral malaria immunity.

*Plasmodium falciparum* is the most virulent of the human malaria species and has adapted to sequester in the host microvasculature [1], leading to organ complications when infected erythrocytes (IEs) accumulate in the microcirculation of the brain (cerebral malaria) or placenta (placental malaria) [2, 3]. Cerebral malaria kills hundreds of thousands of children each year [4] and leaves others with neurological disabilities or cognitive deficits [5]. In highly malaria endemic regions, the primary burden of severe disease occurs in children under 5 years old [4]. Clinical immunity increases with age and exposure to *P. falciparum* reaching a status of premunition where individuals rarely develop cerebral malaria, but the mechanisms of protection are incompletely understood [6].

Parasite cytoadhesion is mediated by a large and diverse protein family, called *Plasmodium falciparum* endothelial membrane protein 1 (PfEMP1) [7]. By switching between the expressed PfEMP1 variant at the IE surface, parasites evade antibodies and alter binding properties [1, 7]. PfEMP1 encode multiple adhesion domains, called the Duffy-binding like (DBL) and cysteine-rich interdomain region (CIDR) [8, 9]. Most PfEMP1 encode an N-terminal head structure, consisting of a DBL and a CIDR domain [8]. Head structures have diversified for binding to CD36 (CIDRα2–6 domains) [10, 11], endothelial protein C receptor (EPCR) (CIDRα1 domains) [12, 13], or unknown binding properties (CIDRβ, γ, or δ domains) [7]. The EPCR-binding subset is linked to severe malaria and parasite binding to brain endothelial cells [13–19].

Given the importance of sequestration in *P. falciparum* pathology, the role of adhesion-blocking antibodies is of interest in understanding mechanisms of anti-disease immunity [20, 21]. PfEMP1 are the major targets of the host antibody response to the IE surface [22, 23] and antibodies to PfEMP1 are thought to contribute to protective immunity against severe childhood malaria [20]. Studies using recombinant PfEMP1 domains have shown that the breadth of antibody specificity increases with repeated infections [24–26]. Antibodies against the EPCR-binding subset of PfEMP1 variants tend to be acquired early in life in children residing in intense malaria transmission settings and are boosted by severe malaria infections [24, 25, 27–30]. However, the functional activities of these antibodies are unknown. Research on malaria during pregnancy has shown that anti-adhesion antibodies play an important role in preventing *P. falciparum*-IE sequestration in the placenta [21, 31–33], but little is known about the role of adhesion-blocking antibodies in cerebral malaria due to the inaccessibility of the brain microvasculature in life and the lack of a *P. falciparum* cerebral malaria animal model.

Recently, we have developed a perfusable 3-dimensional (3D) human brain microvessel platform for modeling the binding interaction of *P. falciparum*-IEs with human brain endothelial cells under dynamic flow conditions [34]. To gain molecular insight into the role of anti-adhesion antibodies in cerebral malaria, we screened the plasma of teenagers and young adults in Malawi on *P. falciparum*-IEs by flow cytometry to assess surface reactive antibodies and used 3D human brain microvessels to measure adhesion blocking. Our study provides evidence that older Malawian children and young adults develop adhesion-blocking antibodies that reduce parasite binding in brain vessels.

## METHODS

### Study Population

Human subjects were enrolled as part of a longitudinal cohort study in Southern Malawi [35]. Because cerebral malaria is extremely rare in children over 9 years old living at our study site [36], we selected samples from individuals between the age of 9 and 29 years old. Venous blood samples were collected in 2021 and stored at −80°C after plasma separation. These individuals were malaria negative at the time of sample collection but have had at least 1 episode of malaria (rapid diagnostic test [RDT]-positive with RDT done by a study nurse during passive case detection) within the prior year and a half.

### Ethical Approval and Consent to Participate

This study was approved by the Institution Review Board of the Malawi National Health Science Research Committee, Michigan State University, Seattle Children's Research Institute, and University of Maryland. Written informed consent was obtained from adults and guardians on behalf of children <18 years old. Assent was also obtained from children 13 years and older.

### *P. falciparum* Culture

This study used 2 Malawian cerebral malaria isolates (2950 and 2742), 2 asymptomatic malaria isolates (5232 and 5196), and *P. falciparum* laboratory line IT4var19 [14]. Parasites were maintained in human red blood cells (O+) and 10% pooled human A+ serum rich RPMI 1640 medium at 37°C. Synchronized asexual *P. falciparum* cultures were obtained by treatment with 5% D-sorbitol (Sigma Aldrich) and by passing the IT4var19 parasite culture through magnetic activated cell sorting (MACS) columns (LD columns; Miltenyi Biotec) for trophozoite enrichment.

### Flow Cytometry

Trophozoite-stage *P. falciparum*-IEs (5% parasitemia) were stained with SYBR green DNA stain (Thermo Fisher) at 1:5000 and incubated with 1:10 dilution plasma samples for 1 hour at 37°C. Cells were then washed, followed by incubation with goat anti-human IgG Alexa Fluor 647 (Invitrogen) at a 1:200 dilution in 1× phosphate-buffered saline (Corning) plus 0.5% bovine serum albumin. Flow cytometry was conducted with a CytoFLEX analyzer (Beckman Coulter) for the Malawi field isolates and an LSR II flow cytometer (BD Biosciences) for IT4var19. The mean fluorescence intensity (MFI) of Alexa Fluor 647 was obtained from 10 000 SYBR green-positive *P. falciparum*-IEs. Data was analyzed using FlowJo (10.8.1 TreeStar, Inc).

### Human Brain Microvascular Endothelial Cell Culture

Primary human brain microvascular endothelial cells (HBMECs; Cell Systems, ACBRI 376, passage 3) underwent expansion and culture following the manufacturer's guidelines. HBMECs were cultured in a tissue culture flask (Corning) in EGM-2 Basal Medium (Lonza) supplemented with EGM-2 MV Microvascular Endothelial Cell Growth Medium SingleQuots (Lonza) and incubated at 37°C with 5% CO_2_. Cells were maintained as a monolayer until seeded in 3D microvessels up to passage 5 to 6.

### 3D Brain Microvessel Fabrication

Three-dimensional brain microvessels were fabricated with a 13 × 13 network geometry using 7.5 mg/mL collagen type I, as previously described [34, 37]. In brief, the fabrication process involved assembling top and bottom collagen layers to create an acellularized, perfusable network with inlet and outlet ports. Then, 200 000 HBMECs were seeded into the acellular channels via both directions and cultured for 3–5 days at 37°C with 5% CO_2_ to form a confluent lumen. The EGM2-MV cell growth medium was refreshed twice daily under gravity-driven flow via the inlet. 3D brain microvessels underwent a visual quality inspection under phase microscopy and any microvessel exhibiting delamination of endothelial cells from the collagen matrix were excluded from the perfusion experiments.

### Perfusion of 3D Brain Microvessels

For perfusion studies, MACS-enriched, trophozoite-stage IT4var19-IEs (DC8-EPCR binder) were divided into 2 equal portions and each portion was membrane labeled using either fluorescent cell linker kit PKH67 green (Sigma) or PKH26 red (Sigma), following the manufacturer's instructions. The stained IEs were resuspended in EGM-2MV medium at 5 × 10^6^ cells/mL. Green-labeled IEs were incubated with Malawi plasma at a 1:10 dilution (plasma exposed) and red-labeled IEs remained in EGM-2MV medium (plasma nonexposed) for 1 hour at 37°C, followed by washing 1 time with EGM-2MV growth medium. Cells were then recounted with a hematocytometer and an equal amount of 2.5 × 10^6^/mL red (plasma nonexposed) and green (plasma exposed)-stained IEs was combined (total = 5 × 10^6^/mL). A 150-µL aliquot of the IE mixture was introduced to the inlet and 50 µL EGM-2MV medium to the outlet for gravity-driven flow. After 1 hour perfusion, medium from the inlets and outlets were discarded, and each microvessel was washed with fresh EGM2-MV growth medium twice for a total of 30 minutes to remove unbound IEs. Microvessels were then fixed with 3.7% paraformaldehyde and 0.008% glutaraldehyde in 1× Dulbecco's phosphate-buffered saline (DPBS) for 15 minutes at room temperature, followed by three 10-minute washes with 1× DPBS. To identify IEs and endothelial cells, microvessels were stained with 1:250 Hoechst (Thermofisher) in 1× DPBS for 30 minutes at room temperature in the dark, followed by three 1× DPBS washes for 10 minutes each. For EPCR blockade, 3D brain microvessels were pretreated with 50 μg/mL rat anti-human EPCR monoclonal antibody (Sigma-Aldrich clone 252) for 1 hour before perfusing with fluorescent membrane-labelled *P. falciparum*-IEs at 5 × 10^6^/mL. The anti-EPCR antibody-treated vessels underwent the same downstream processing as above.

### Parasite Binding Quantification

Fixed and stained microvessels were imaged using a Nikon TiE inverted widefield microscope with images acquired at a 5–10 µm z-step size, which gave sufficient z-resolution required for parasite counting. IE binding was assessed by counting attached red blood cells exhibiting double-positive, membrane labeling (red or green) and parasite nuclear stain across all optical slices using Fiji software (ImageJ, version 1.5.4). The multipoint selection tool in ImageJ was employed to assign labels for the 2 counter types of IEs: green fluorescent (plasma-exposed IT4var19-IEs) and red fluorescent (nonexposed IT4var19-IEs). The total number of IEs from both red and green populations was quantified along the bottom lumenal wall across the entire 13 × 13 grid network (total = 312 branches). A second counting strategy only considered bound cells along the first (column 1) and the last (column 13), facilitating quantification of flow-dependent binding inhibition at different estimated wall shear stress, as described [34]. Heatmaps were generated to display the digitized averaged binding distribution from biological replicates, as previously described [38]. The percentage of adhesion-blocking activity was calculated as the proportion of bound green (plasma-exposed IT4var19-IEs) versus bound red (nonexposed IT4var19-IEs). Percentage inhibition was averaged across all replicates (n = 3 to 5 independent microvessels per plasma sample).

### Wall Shear Stress Numerical Simulation

The initial fluid flow characteristics in the 3D brain microvessels before medium level equilibration were simulated using COMSOL Multiphysics software, package version 6.0. Considering the effect of gravity-driven flow in small-diameter microchannels and a perfusate with low hematocrit, the flow was assumed to be unidirectional, laminar, and Newtonian. The analysis was solved using predefined Navier-Stokes equation in COMSOL with the stationary solver and applying no slip boundary condition and no fluid leakage through the wall. Fluid properties were defined as follows: viscosity of 10^−3^ Pa·s and density of 10^3^ kg/m^3^ at 293.15 K. Initial conditions were specified with a static pressure of 87.770 Pa (equivalent to 0.895 cm H_2_O) exerted by a column height of 150 µL perfusate at the inlet and 15.098 Pa exerted by a 50 µL perfusate at the outlet. Wall shear stress was calculated by averaging values from the centerline adjacent to each collagen square grid across column 1 at z = 0. The results were imported into and analyzed using MATLAB R2018a. The solution reported was mesh independent, verified by a convergence of averaged wall shear stress within 5% differences between 2 mesh sizes.

### Statistical Analysis

Statistical analyses were conducted using GraphPad Prism software (version 10.0.3, Graphpad Software, Inc). Statistical significance of differences between bound IEs populations (plasma exposed vs nonexposed) was assessed using the unpaired *t* test. For scatter plots illustrating linear relationships between variables, nonparametric Spearman correlation analysis was used.

## RESULTS

### Study Participants

Plasma samples were collected at the final visit of a cohort study in Southern Malawi [35]. Plasma from a total of 48 individuals, including 32 school-age children (9–15 years) and 16 young adults (16–29 years) were included in this study. Study participants had 1–6 episodes of RDT-positive malaria within the past 1.5 years and infection prevalence was higher in younger children (Table 1).

**Table 1.** Demography of Plasma Donors and History of Malaria Infection

Characteristics	No. (Male/Female)	Age, Mean ± SD
Donor age, y		
9–15	32 (17/15)	…
16–29	16 (4/12)	…
Malaria episodes		
1	25 (10/15)	15.8 ± 5.8
2	7 (2/5)	16.5 ± 5.92
3	9 (5/4)	14.3 ± 5.67
4	5 (3/2)	10.8 ± 1.1
5	1 (0/1)	11
6	1 (1/0)	9

### Reactivity of Plasma From Malawian Older Children and Young Adults With *P. falciparum*-IEs

**Figure 1.**
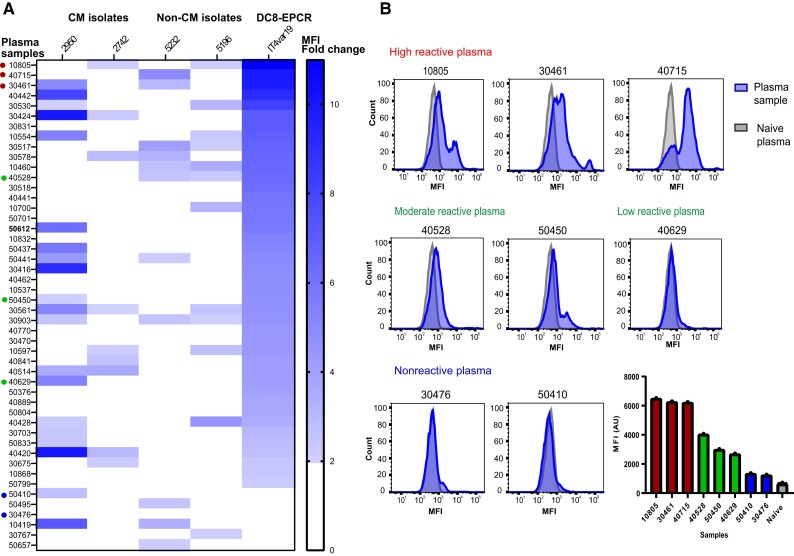
Plasma reactivity of older children and young Malawian adults to local parasite isolates and a DC8-EPCR–binding parasite line. *A*, Flow cytometric recognition of *Plasmodium falciparum*-IEs by plasma samples from 48 Malawian individuals done in duplicate. Heat map shows plasma reactivity with cerebral malaria isolates (2950 and 2742), noncerebral malaria isolates (5232 and 5196), and the IT4var19 (DC8-EPCR) parasite line. Reactivity is calculated as the ratio of the MFI obtained from plasma samples, relative to that of the negative control (US plasma) and expressed as fold change. Plasma sample reactivities < 2-fold changes were considered negative. *B*, Representative histograms of 8 different plasma samples to IT4var19-IEs chosen for adhesion-blocking tests in 3D brain microvessels, ranging from high reactive, low to moderate reactive, and nonreactive. Bar chart shows the flow cytometry MFI values obtained from the plasma samples. Abbreviations: 3D, 3 dimensional; AU, arbitrary unit; CM, cerebral malaria; EPCR, endothelial protein C receptor; IE, infected erythrocyte; MFI, mean fluorescence intensity.

Malawian plasma samples were analyzed by flow cytometry on *P. falciparum*-IEs using 4 local parasite isolates and IT4var19, a clonally variant DC8-EPCR–expressing parasite line that was selected in vitro on human brain endothelial cells [14]. IT4var19 was confirmed to predominately expresses a single *var* transcript (Supplementary Figure 1). The plasma sample reactivity was analyzed in relationship to naive plasma pool (US donors) and expressed as its ratio (patient sample/naive plasma pool) (Figure 1*A*). For 2 cerebral malaria isolates (2950 and 2742), we observed surface reactivity (defined as ≥ 2-fold change) by 42% and 19% of plasma samples, respectively. For 2 asymptomatic parasite isolates (5232 and 5196), we observed surface reactivity by 23% and 25% of plasma samples, respectively. By comparison, over 90% of plasma samples reacted with IT4var19-IEs (Figure 1*A*). Consistent with studies conducted with recombinant CIDR domains showing that antibody responses tend to increase over early childhood and then plateau in teenage years [30], many of the highest antibody responses to the 4 parasite field isolates were found in children between 9 and 15 years old and there was an age-dependent increase in antibody response against IT4var19 (*r* = 0.347, *P* = .01 Spearman correlation) (Supplementary Figure 2).

### Malawian Plasma Inhibits Binding of *P. falciparum*-IEs in a 3D Brain Microvessel Model

**Figure 2.**
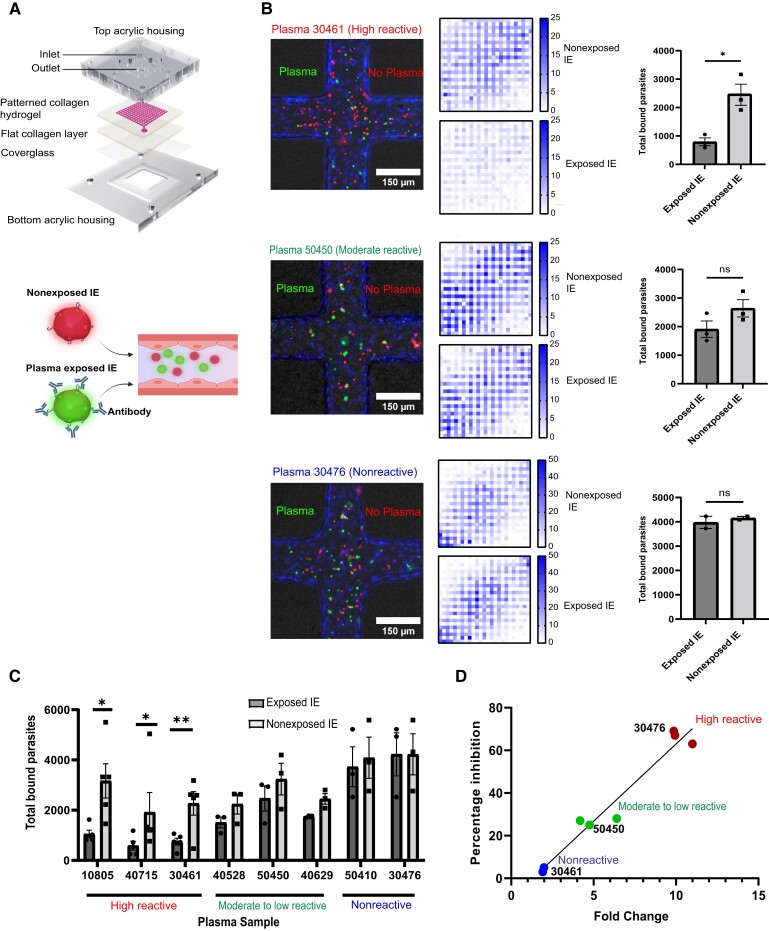
Adhesion-blocking properties of plasma from teenagers and young adults in 3D brain microvessels. *A*, Schematic of a 3D brain microvessel device showing the 13 × 13 patterned collagen hydrogel. For adhesion-blocking studies, equal numbers of Malawi plasma exposed IEs (green fluorescent membrane stain) and non-plasma exposed IEs (red fluorescent membrane stain) were co-perfused in the microvessels. The blood vessel schematic was created with BioRender.com. *B*, Binding of plasma-exposed IEs and non-plasma exposed IEs. The Hoechst nucleic acid stain shows the nuclei of the endothelial cells lining the lumen wall. Heat map shows the mean number of bound IEs across the 13 × 13 grid in the presence and absence of Malawi plasma (n = 5 independent 3D brain microvessel devices for high reactive plasma samples, n = 3 independent 3D brain microvessel devices for moderate to low and nonreactive plasma samples). Bar charts show the total bound IEs of plasma exposed and nonexposed microvessels (error bars indicate standard error of mean; *P < .05, unpaired *t* test). *C*, Bar charts shows the total bound IEs of plasma exposed and non-plasma exposed for each plasma sample tested (n = 5 independent 3D brain microvessel devices for high reactive plasma samples, n = 3 independent 3D brain microvessel devices for moderate to low and nonreactive plasma samples; *P < .05, **P < .01, unpaired *t* test). *D*, Correlation of the binding inhibitory activity of each plasma in 3D brain microvessels and reactivity to IT4var19-IEs (MFI fold change). Abbreviations: 3D, 3 dimensional; IE, infected erythrocyte; MFI, mean fluorescence intensity; ns, not significant.

To investigate anti-adhesion activity of the Malawian plasma samples, we perfused IT4var19-IEs in the 3D human brain microvessel platform [34]. This parasite line is representative of the DC8-EPCR–binding subset that is elevated in pediatric cerebral malaria patients and linked to severe brain swelling in Malawi [17]. Our 3D brain microvessels have a characteristic channel lumen of approximately 100–120 µm within a 13 by 13 grid network geometry (Figure 2*A*). The grid layout creates a range of flow velocities within a single microvessel device, which offers a platform to study the flow dependency of *P. falciparum*-IE binding and antibody effector functions.

We selected 8 Malawian plasma samples that ranged in flow-cytometry reactivity against IT4var19 parasites from highly reactive (plasma 10805, MFI fold-change = 10.9; plasma 40715, MFI fold-change = 9.93; plasma 30461, MFI fold-change = 9.8), to moderate or low reactivity (plasma 40528, MFI fold-change = 6.4; plasma 50450, MFI fold-change = 4.7; plasma 40629, MFI fold-change = 4.1), and to nonreactive samples (plasma 30476, MFI fold-change = 1.90; plasma 50410, MFI fold-change = 1.98) (Figure 1*B*). To assess adhesion-blocking activity of plasma samples, we co-perfused the 3D brain microvessels with an equal mixture of enriched trophozoite-stage IT4var19-IEs using 2 different color-stained fluorescent dyes to distinguish between plasma exposed (green fluorescent stained) or nonexposed (red fluorescent stained) (Figure 2*A*). After 1 hour perfusion and washing of unbound cells, we quantified the number of red and green IEs across the grid and converted it to a heat map (Figure 2*B*). Binding of IT4var19-IEs was flow dependent with decreased binding in the 2 corners near the inlet and the outlet, where the flow rates are higher (Figure 2*B*). For the 3 highly reactive plasma samples, we observed substantially reduced IT4var19-IE binding in the 3D brain microvessels (plasma 10805, 63% inhibition; plasma 30461, 69% inhibition; plasma 40715, 67% inhibition; Figure 2*B* and 2*C*). By comparison, there was lower inhibitory activity of the low to moderately reactive plasma samples (plasma 40528, 28% inhibition; plasma 40629, 27% inhibition; plasma 50450, 25% inhibition) and negligible inhibition with nonreactive plasma samples (plasma 50410, 5% inhibition, plasma 30476, 3% inhibition; Figure 2*B* and 2*C*). Overall, there was a positive correlation between plasma reactivity to IT4var19-IEs by flow cytometry and anti-adhesion activity in 3D brain microvessels (Figure 2*D*).

### Impact of Flow Velocity on Plasma Inhibitory Activity in 3D Brain Microvessels

**Figure 3.**
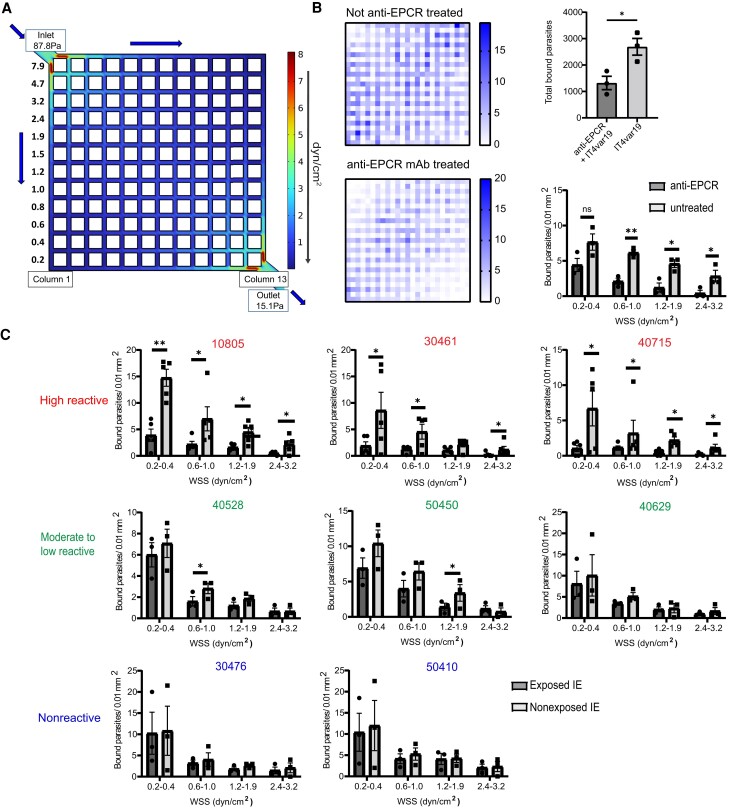
Effect of flow velocity on the adhesion-blocking activity of Malawian plasma in 3D brain microvessels. *A*, COMSOL simulation of the initial estimated WSS (z = 0 μm) distributions of the 13 × 13 3D brain microvessels showing column 1 and column 13 used to study flow-dependency of plasma inhibitory activity. *B*, Binding of IT4var19-IEs in the presence or absence of anti-EPCR monoclonal antibody. Left, heat map of IE binding in 3D brain microvessels that were not treated (top) or pretreated (bottom) with anti-EPCR monoclonal antibody. Right, top chart shows total count of IT4var19-IEs in the vessels. Bottom chart shows quantification strategy considering only column 1 and 13 for effect of flow velocity on adhesion inhibition (error bars indicate standard error of mean). *C*, Flow-dependent inhibitory activity of Malawi plasma samples considering columns 1 and 13 to assess binding inhibition at different flow regions in the microvessels (n = 5 independent 3D brain microvessel devices for high reactive plasma samples, n = 3 independent 3D brain microvessel devices for moderate to low and nonreactive plasma samples; *P < .05, **P < .01, unpaired *t* test). Abbreviations: 3D, 3 dimensional; EPCR, endothelial protein C receptor; IE, infected erythrocyte; mAb, monoclonal antibody; WSS, wall shear stress.

Because the blood flow rate differs between microvascular sites where IEs can sequester [39], we also assessed the relationship between flow velocity and plasma binding inhibition. For this analysis, we quantified IE binding inhibition along the first and last channels in the 13 × 13 microvessel grid (Figure 3*A*). In the first channel of the microvessel device, there is a descending flow rate that is highest near the inlet and lowest in the bottom corner. In the last channel, there is an ascending flow rate that is lowest in the upper left corner and highest near the outlet (Figure 3*A*). Initially, we studied binding inhibition with an EPCR monoclonal antibody. For these experiments, the 3D brain microvessels were pretreated with anti-EPCR (mAb 252) for 1 hour and washed with fresh medium prior to perfusion with IT4var19-IEs. As expected, binding of IT4var19-IEs was flow-dependent in the absence of EPCR blockade (Figure 3*B*). We observed over 50% binding inhibition of enriched trophozoite-stage IT4var19-IEs in the vessels (Figure 3*B*), confirming EPCR to be an important receptor in the 3D brain microvessel model [34]. The extent of IE binding inhibition by EPCR blockade varied by flow velocity and was weakest at the lowest flow region of the grid (Figure 3*B*).

Using Malawian plasma, we observed a difference between the 3 highly reactive plasma samples and plasma samples with low to moderate surface reactivity. Whereas highly reactive plasma samples exhibited inhibitory activity at different flow regions across the microvessels, plasma samples with low or moderate reactivity either lacked inhibitory activity or had partial inhibitory activity at moderate flow rates (Figure 3*C*).

## DISCUSSION

Diverse infectious pathogens interface with the blood-brain barrier, but it is challenging to model antibody effector mechanisms at this site [40, 41]. Most previous in vitro models of the human blood-brain barrier for pathogen research have used cell monolayers and have not considered flow dynamics [41], even though microfluidic dynamics are likely to have an important effect on the pathogen-vessel wall interaction and potentially antibody effectiveness. While previous research has established that anti-adhesion antibodies play an important role in protective immunity to block IE sequestration in the placenta [2, 21, 31–33], much less is understood about adhesion-blocking antibodies in cerebral malaria. Here, we used a bioengineered 3D human brain microvessel model that mimics small blood vessels where *P. falciparum*-IEs sequester, to investigate adhesion-blocking antibody responses.

In this study, we focused on children over 9 years old because they have developed immunity to severe disease and are rarely seen in our pediatric cerebral malaria ward [36]. Previous research has indicated that PfEMP1 proteins are immunodominant targets of antibodies [22, 23] and that antibody breadth broadens with age [25, 29, 30, 42, 43]. In line with these findings, our results indicate that older children and young adults residing in Malawi have developed strain-specific antibodies that recognize to different extents local parasite isolates, including both cerebral and noncerebral parasite isolates. Using a DC8-EPCR–expressing parasite line that was selected in vitro on human brain endothelial cells [14] and typifies a subset of EPCR-binding variants that was linked to severe brain swelling in our pediatric cerebral malaria population in Southern Malawi [17], we observed a strong correlation between the level of flow cytometric recognition of IEs by plasma and adhesion-blocking capacity in 3D brain microvessels.

In vivo, blood flow forces vary across the microcirculatory system and flow rates are known to influence blood cell interactions with the vessel wall [44, 45]. The blood flow also varies substantially between parasite sequestration sites, such as the placenta and the brain microcirculation [39, 46] and sequestered IEs markedly alter flow dynamics [47]. However, less is known about how flow dynamics may influence antibody effector functions to prevent *P. falciparum*-IE binding in the brain microcirculation. We found that highly surface reactive plasma exhibited robust inhibitory activity across flow rates, while plasma with low or moderate surface reactivity had limited or no inhibitory activity. This finding indicates there may be a threshold of surface antibody reactivity necessary to achieve robust inhibitory activity across the physiological range of flow rates in the brain microcirculation. Further research is needed to understand whether moderately reactive plasma may have partial inhibitory activity at some blood flow rates. Overall, these data provide evidence that older Malawian children acquire functional antibodies that can inhibit *P. falciparum*-IE binding in 3D brain microvessels and reveals that the quality of the anti-cytoadhesion antibody response is sensitive to antibody titer and flow dynamics.

EPCR-binding PfEMP1 variants have been subclassified into group A and DC8 subtypes [7, 13]. A recent cryo-electron microscopy study suggests that a group A PfEMP1 head structure undergoes a large conformational change upon EPCR binding [48], raising the possibility that the packing of domains may represent a conformational masking strategy to avoid antibodies. In this study, we used a parasite line expressing a DC8-EPCR–binding PfEMP1 variant, which has a chimeric B/A head structure [49]. We found that precoating of *P. falciparum*-IEs with plasma inhibited binding in 3D brain microvessels, suggesting that some antibodies can overcome the conformational masking strategy or alternatively that DC8-EPCR–binding head structures do not form the same compact head structure as group A EPCR binders. In future studies, it will be informative to determine the targets of antibodies that inhibit *P. falciparum*-IE accumulation in 3D brain microvessels.

A limitation of the study is the bioengineered 3D brain microvessel model is labor intensive, which constrains the number of plasma samples that can be evaluated. In addition, because the microvessels are made within a soft collagen biomatrix, small variation in vessel dimensions may affect local flow characteristics. To overcome intervessel variability, we performed head-to-head comparisons of plasma-coated and nonplasma IE controls in the same device. Overall, the ability of a single 3D brain microvessel device to simultaneously assess binding across multiple flow rates can be leveraged to better understand pathogen and antibody effector mechanisms at the blood vessel wall and potentially to evaluate therapeutic antibodies against brain-tropic pathogens.

In summary, this study provides evidence that naturally acquired antibodies can inhibit *P. falciparum*-IE binding in 3D brain microvessels. This finding has implications for clinical immunity that protects against cerebral malaria.

## Supplementary Data

Supplementary materials are available at *The Journal of Infectious Diseases* online (http://jid.oxfordjournals.org/). Supplementary materials consist of data provided by the author that are published to benefit the reader. The posted materials are not copyedited. The contents of all supplementary data are the sole responsibility of the authors. Questions or messages regarding errors should be addressed to the author.

## Supplementary Material

jiae315_Supplementary_Data

## Notes

***Acknowledgment.*** We thank Chifundo Duster for help with field work.

***Author contributions.*** F. J., Y. Z., and J. D. S. designed the study. F. J. and R. H. performed and analyzed 3D microvessel experiments. F. J. and A. S. performed plasma reactivity to field isolates. L. M. C. and K. B. S. conducted the field work. F. J. and J. D. S. drafted initial manuscript. All authors read, edited, and approved the final manuscript.

***Disclaimer.*** The funder had no role in the study design, data collection and interpretation, or decision to submit the work for publication.

***Financial support*.** This work was supported by the National Institutes of Health (grant numbers RO1 AI141602 to J. D. S., K. B. S., and Y. Z.; RO1 AI148802 to J. D. S. and Y. Z.; and U19AI129388 to L. M. C.).
