# Supplementary material for: Plasma From Older Children in Malawi Inhibits Plasmodium falciparum Binding in 3-Dimensional Brain Microvessels
Source: J Infect Dis. 2024 Jun 14;230(6):e1402–11. doi: 10.1093/infdis/jiae315 (PMC11646604; doi:10.1093/infdis/jiae315)
Supplement: jiae315_Supplementary_Data [file jiae315_supplementary_data.zip › SupplementalFigure1_v2.pdf]

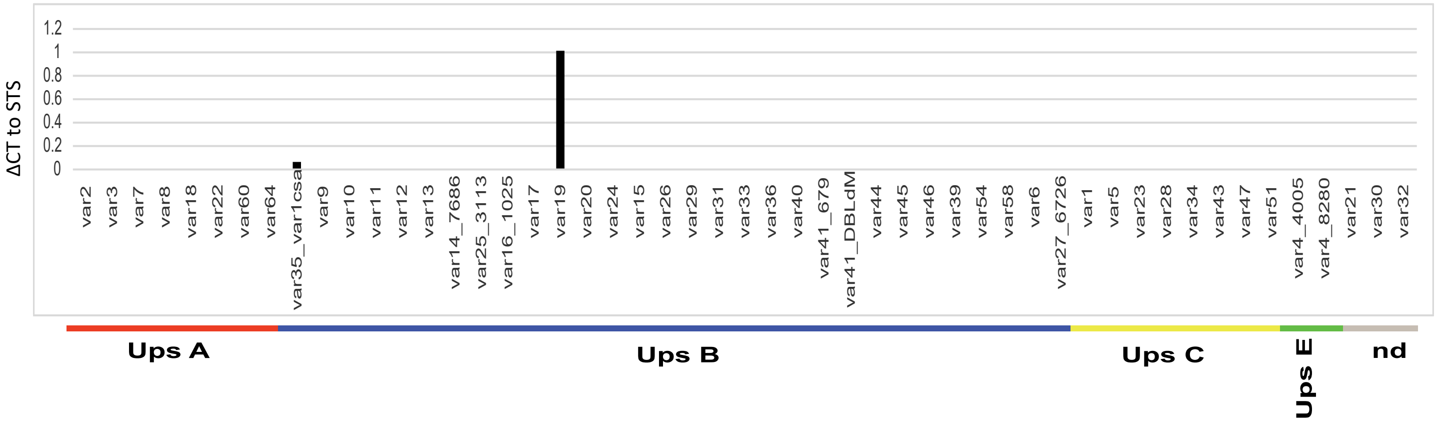

Figure S1. *Var* transcriptional profiling of the IT4var19 parasite line. Transcription profiling was done on ring stage-IEs using qRT-PCR. Transcript levels were expressed relative to the housekeeping gene seryl-tRNA synthetase (STS). Ups: upstream; nd: not determined.
