# Supplementary material for: Plasma From Older Children in Malawi Inhibits Plasmodium falciparum Binding in 3-Dimensional Brain Microvessels
Source: J Infect Dis. 2024 Jun 14;230(6):e1402–11. doi: 10.1093/infdis/jiae315 (PMC11646604; doi:10.1093/infdis/jiae315)
Supplement: jiae315_Supplementary_Data [file jiae315_supplementary_data.zip › SupplementalFigure2_v2.pdf]

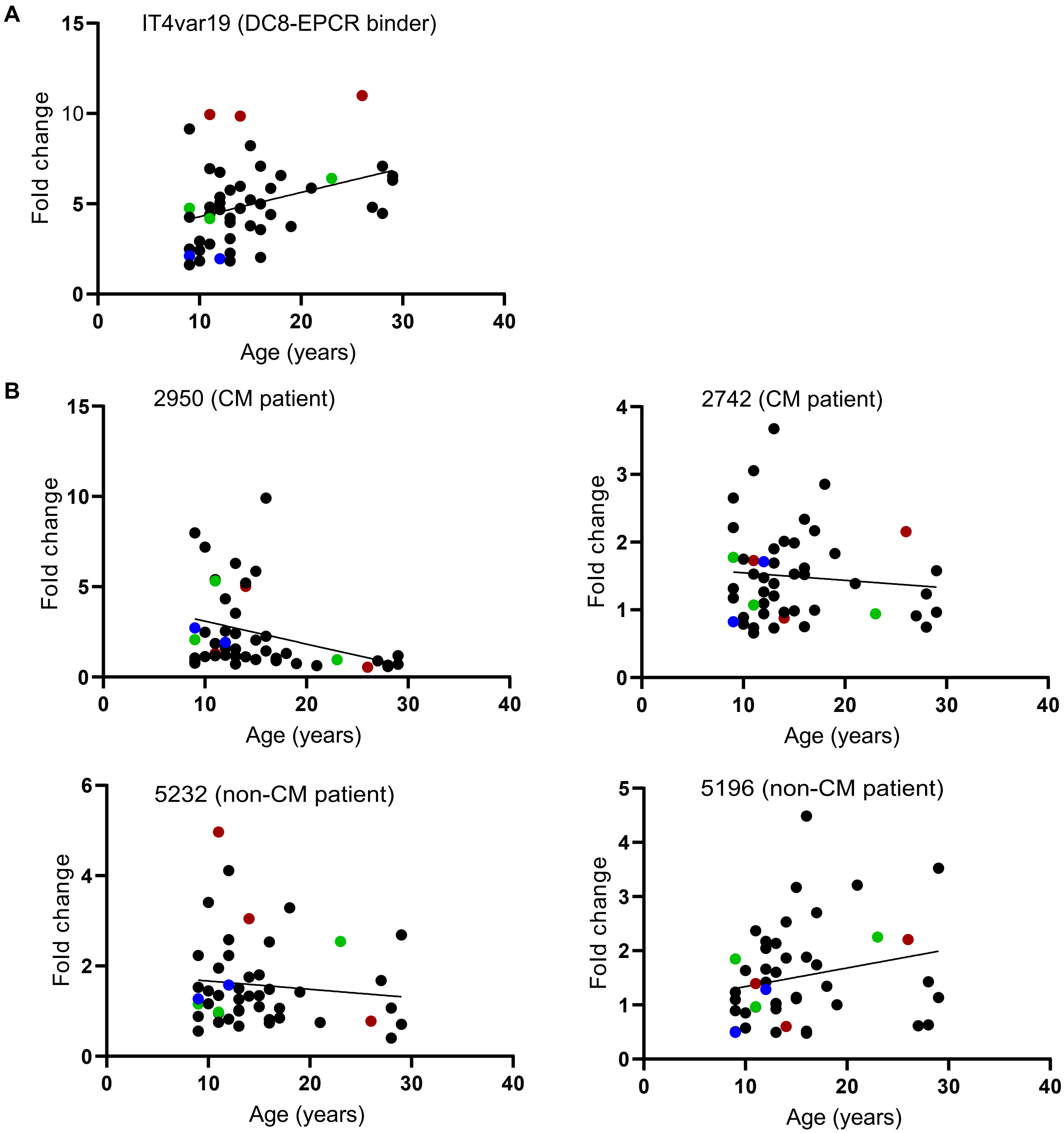

Figure S2. Correlation of reactivity to different parasite isolates with the age of plasma donors (A) Scatter plots show correlation of the reactivity of plasma samples to IT4var19 (DC8-EPCR) and the age of the plasma donor. Plasma samples highlighted in red, green, and blue represent high, low to moderate, and non-reactive samples respectively. Spearman's correlation coefficient ($r = 0.347$; $p=0.01$). (B) Scatter plots show correlation of the reactivity of plasma samples to four local Malawian parasite isolates with the age of the plasma donors. Spearman's correlation coefficient (isolate 250 $r= -0.333$; $p= 0.02$, isolate 2742 $r= -0.096$; $p= 0.51$, isolate 5232 $r= -0.111$; $p=0.45$, isolate 5196 $r= 0.226$; $p= 0.12$).
